# Influence of obesity on the short-term outcome of laparoscopic colectomy for colorectal cancer

**DOI:** 10.4103/0972-9941.37192

**Published:** 2007

**Authors:** Kazuhiro Sakamoto, Shinichiro Niwa, Masanobu Tanaka, Michitoshi Goto, Hironobu Sengoku, Yuichi Tomiki

**Affiliations:** Department of Coloproctological Surgery, Juntendo University, School of Medicine, Tokyo, Japan

**Keywords:** Colorectal cancer, laparoscopic colectomy, obesity

## Abstract

**Purpose:**

Obesity has been generally associated with increased surgical risk. However, data on the outcome of laparoscopic colectomy in obese and non-obese patients are controversial. The aim of this study is to assess the short-term outcome of laparoscopic colectomy for colorectal cancer (CRC) in obese patients as compared with non-obese patients.

**Materials and Methods:**

Sixty-nine patients who underwent laparoscopic anterior resection for CRC during the past six years were retrospectively evaluated. The patients with CRC involving the sigmoid or rectosigmoid colon and subjected to intracorporeal anastomosis were included in this study. They were divided into three groups according to body mass index (BMI): obese (BMI ≥ 28.0 kg/m^2^), pre-obese (BMI: 25.0-27.9 kg/m^2^) and non-obese (BMI < 25.0 kg/m^2^).

**Results:**

Nine patients (13.0%) were obese, 11 patients (15.9%) were pre-obese and 49 patients (71.1%) were non-obese. Patient characteristics, such as age, gender, tumor location, previous laparotomy, were similar among the three groups. There were no significant differences in operative time, blood loss, intraoperative complications and conversion rates. Postoperative complications and duration of postoperative hospital stay were also similar among the three groups. However, two of the three patients in the pre-obese group had to be operated on again due to incarceration of the small bowel into a port site.

**Conclusions:**

Laparoscopic colectomy can be safely performed in obese patients with short-term results similar to those obtained in non-obese and pre-obese patients.

## INTRODUCTION

The rates of obesity are very high at present in Western countries. The rate has also increased gradually in Asian countries, including Japan. However, the percentages of obese people in Asian populations are lower than those in Western populations.[1] Obesity relates to various diseases and may be associated with increased risk of cancers.[2,3] In general surgery, it has been considered one of the risk factors.[4] In laparoscopic surgery, it has been considered that obesity may reduce technical feasibility, prolonging operative time and increasing operative blood loss and has been regarded as a relative contraindication factor for laparoscopic surgery.[5–7] Recently, with the improvement of laparoscopic techniques and instruments, laparoscopic surgery has been proposed as a promising approach for obese patients. However, the outcome of laparoscopic colectomy in obese patients is controversial. Indeed, some investigators have suggested that laparoscopic colectomy for diverticular diseases and colorectal cancer (CRC) can be performed safely in obese patients,[8,9] while others have reported higher rates of conversions and complications than in non-obese patients.[5,10]

Laparoscopic anterior resection is a standard procedure in laparoscopic colectomy. However, this procedure generally requires several laparoscopic techniques, including dissection of the rectosigmoid mesentery, ligation of the major vessels and intracorporeal anastomosis using a circular stapler. The aim of this study is to assess the short-term outcome of laparoscopic colectomy for CRC in pre-obese and obese patients as compared with non-obese patients.

## MATERIALS AND METHODS

All patients who underwent laparoscopic anterior resection for CRC in our hospital between January 2001 and June 2006 were retrospectively evaluated. The patients with CRC involving the sigmoid (S) or rectosigmoid (RS) colon and subjected to intracorporeal anastomosis using the Double Stapling Technique (DST) were included in this study. Patients who underwent D1 lymph node dissection[11] were excluded. Body mass index (BMI height and body weight; kg/m^2^) was used as an objective measure of obesity. Accordingly, patients were classified into three groups; obese (BMI ≥ 28.0 kg/m^2^), pre-obese (BMI: 25.0-27.9 kg/m^2^) and non-obese (BMI < 25.0 kg/m^2^).

The data analyzed included patient characteristics (age, gender, previous laparotomy, BMI and tumor location), intraoperative data (approach method, port number, wound incision, lymph node dissection, operative time, blood loss and intraoperative complications), pathological data (depth of tumor invasion, number of harvested nodes and pathological stage) and short-term outcome (conversion to hand-assisted laparoscopic surgery (HALS) and laparotomy, postoperative complications and length of postoperative hospital stay).

D2 lymph node dissection[11] was defined as the ligation of the inferior mesenteric artery (IMA) below the left colic artery (LCA) and the removal of paracolic and intermediate nodes. Therefore, for this analysis we included patients in whom the IMA was ligated below the LCA after circumferential or partial dissection around the trunk of the IMA. Intraoperative complications were defined as conditions that needed additional treatments intra or extracorporeally, from a tiny serosal bowel injury due to burning with an electric forceps to bleeding resulting in conversion to laparotomy.

Tumor location, staging and lymph node dissection were defined according to the Japanese Classification of Colorectal Carcinoma (7^th^ edition).[11]

### Laparoscopic surgical procedure

In all cases, a pneumoperitoneum was established after the open insertion of a 12-mm port below the umbilicus. Subsequently, four additional ports were placed as usual; a 12-mm port in the right lower quadrant, a 10-mm port in the right flank and one 5-mm port each in the left lower quadrant and the left flank. Additional ports (5-mm) were used when necessary. The pneumoperitoneum pressure was kept at 8-10 mmHg. We followed two approaches: namely, the lateral-to-medial (lateral) and medial-to-lateral (medial) approach. For the lateral approach the lateral peritoneal attachments were dissected first and then the rectosigmoid mesentery was explored and the proximal vessels (on the trunk of IMA or below of the LCA) were ligated. In contrast, for the medial approach the proximal vessels were initially explored and divided: the rectosigmoid mesentery was dissected at the base and then laterally. The splenic flexure was mobilized when necessary. The distal end of the rectum was resected with a laparoscopic stapler. The 12-mm port of the umbilicus was removed and the incision was enlarged. The colon was then extracted through this incision and the anvil of a circular stapler was inserted in the proximal colon. The pneumoperitoneum was reestablished and intracorporeal anastomosis was performed using DST.

### Statistical analysis

Statistical analysis was performed with the Statistical Package for the Social Science (SPSS) statistical program (Dr. SPSS 2 for Window, SPSS Japan Inc. Tokyo). Statistical comparison was performed using the Chi-square test, Kruskal-Wallis and Mann-Whitney test, as appropriate. A *P* value <0.05 was set as the statistically significant level.

## RESULTS

Between January 2001 and June 2006, 69 patients with CRC involving the sigmoid or rectosigmoid colon underwent laparoscopic anterior resection. The patients were distributed into three groups according to their BMI: except for this parameter, patient characteristics were similar in the groups [Table 1].

**Table 1:** Characteristics of patients in the obese, pre-obese, and non-obese

	Obese (n=9)	Preobese (n=11)	Nonobese (n=49)	*P*-value
Age (ys)				
Mean	60.6 ± 10.0	60.6 ± 8.4	59.8 ± 10.9	
Range	31-71	44-73	31-80	0.103
Gender				
Male	5 (55.6)	8 (72.8)	32 (65.3)	
Female	4 (33.3)	4 (36.4)	17 (34.7)	0.725
Previous laparotomy				
(−)	6 (66.7)	7 (63.6)	32 (65.3)	
(+)	3 (33.3)	4 (36.4)	17 (34.7)	0.990
Body mass index (kg/m^2^)				
Mean	29.8 ± 1.4	26.3 ± 0.9	22.1 ± 1.7	
Range	28.0 - 32.5	25.2 - 27.7	19.0 - 24.9	<0.001
Tumor location				
Sigmoid (S)	4 (44.4)	7 (66.7)	33 (67.3)	
Rectosigmoid (RS)	5 (55.6)	4 (33.3)	16 (32.7)	0.422

Figures in parentheses are in percentage

Operative data are presented in Table 2. There were no significant differences in the approach method, the number of ports used, the degree of lymph node dissection, operative time and blood loss among the three groups. However, the length of the wound incision to extract the colon differed significantly among the three groups (*P*=0.007): this was significantly longer in the obese group than in the non-obese group (*P*=0.003). There was no difference in the rate of intraoperative complications among the three groups (*P*=0.600). Five bowel injuries were thermal burns caused with the electrical forceps. Although none of them reached deeper than the serosal layer, sutures were performed intra or extracorporeally. In two of the 13 patients with an intraoperative complication in the non-obese group, one was converted to HALS because of hemorrhage and the other was converted to open laparotomy because of a hemi-circumferential anastomotic leak that was due to the misfire of the circular stapler. In the obese and the pre-obese groups, none of the patients required conversion to HALS or laparotomy due to intraoperative complications. There was no difference in the rate of conversion to HALS and laparotomy among the three groups (*P*=0.676 and *P*=0.813, respectively) [Table 3].

**Table 2:** Operative data of patients in the obese, pre-obese, and non-obese

	Obese (n=9)	Preobese (n=11)	Nonobese (n=49)	*P*-value
Approach method				
Lateral approach	5 (55.6%)	6 (54.5%)	24 (49.0%)	0.901
Medial approach	4 (44.4%)	5 (45.5%)	25 (51.0%)	
Port number				
Median (range)	5 (5-6)	5 (5-7)	5 (3-7)	0.284
Wood incision (cm)				
Median (range)	4.5 (4.0-5.5)	4.0 (4.0-4.5)	4.0 (3.0-6.0)	0.007
Lymph node dissection				
D2	8 (88.9%)	7 (63.6%)	32 (65.3%)	0.356
D3	1 (11.1%)	4 (36.4%)	17 (34.7%)	
Operative time (min)				
Median (range)	225 (195-342)	245 (185-420)	224 (165-412)	0.112
Blood loss (ml)				
Median (range)	40 (5-270)	26 (5-115)	25 (2-147)	0.576
Intraoperative complication	1 (11.1%)	3 (27.3%)	13 (26.5%)	0.600
Hemorrhage	0	2	6 (1)*	
Bowel injury	1	0	4	
Staple misfire	0	0	1 (1)**	
Pneumoderma	0	1	1	
Other	0	0	1	

(1)* No. of conversion to HALS

(1)** No. of conversion to open surgery

**Table 3:** Reasons for conversions to hand-assisted laparoscopic surgery or laparotomy

	Obese (n=9)	Preobese (n=11)	Nonobese (n=49)	*P*-value
Conversion to hand-assisted laparoscopic surgery	0	1 (9.1%)	3 (6.1%)	0.676
Hemorrhage	0	0	1	
Adhesion	0	0	1	
Unclear anatomy	0	1	1	
Conversion to laparotomy	0	0	1	0.813
Staple misfire	0	0	1 (2.0%)	

The median number of harvested nodes was 10 in the obese group, 12 in the pre-obese and 14 in the non-obese group, with no significant difference among the three groups (*P*=0.305). The distribution of tumor depth and pathological stage were also similar in them [Table 4].

**Table 4:** Pathological data of patients in the obese, pre-opbese and non-obese groups

	Obese (n=9)	Preobese (n=11)	Nonobese (n=49)	*P*-value
Depth of invasion				
m	1 (11.1%)	0	2 (4.1 %)	0.296
sm	7 (77.8%)	5 (45.5%)	34 (69.4%)	
mp	1 (11.1%)	4 (36.4%)	6 (12.2%)	
ss	0	2 (18.2%)	7 (4.3%)	
No. of nodes				
Median (range)	10 (4-16)	12 (2-28)	14 (2-47)	0.305
Pathological stage				
Stage 0	2 (22.2%)	0	3 (6.1 %)	0.517
Stage I	6 (66.7%)	8 (72.7%)	31 (63.3%)	
Stage II	0	2 (18.2%)	4 (8.2%)	
Stage III a	1 (11.1%)	1 (9.1 %)	9 (8.4%)	
Stage III b	0	0	2 (4.1%)	

Data on the short-term outcome of surgery are presented in Table 5. The postoperative complication occurred in three patients (27.3%) in the pre-obese group and six patients (12.2%) in the non-obese group, but there was no postoperative complication in the obese group. The postoperative complication rate was similar among the three groups (*P*=0.188). In the pre-obese group, the two patients who were diagnosed as having ileus, were operated on again within a few days after the first operation: they presented incarceration of the small bowel into a port site. All four wound infections, one in the pre-obese and three in the non-obese group, occurred in the incision below the umbilicus; that is the port through which the surgical specimen had been withdrawn. The median duration of postoperative hospital stay was also similar among the three groups (*P*=0.583) [Table 5].

**Table 5:** Short-term outcome of patients in the obese, preobese and nonobese groups

	Obese (n=9)	Preobese (n=11)	Nonobese (n=49)	*P*-value
Postoperative complication	0	3 (33.3%)	6 (66.7%)	0.188
Ileus	0	2 (2)*	1 (1)*	
Anastomotic leak	0	0	0	
Wound infection	0	1	3	
Enterocolitis	0	0	1	
Other	0	0	1	
Re-operation	0	2 (18.2%)	1 (2.0%)	0.047
Post-operative hospital stay (day)				
Median (Range)	11 (7-19)	12 (9-40)	12 (7-58)	0.583

(1)* No. of re-operation

## DISCUSSION

Obesity has been increasing globally. Indeed, nearly one-third of adults are obese (BMI>30) in the United States.[12] It has also increased gradually among Japanese people. However, the percentage of patients with a BMI > 30 in Asian populations is lower than in Western populations. According to one study on the Japanese statistics showed that 23.9% of males and 30.2% of females were in the category of BMI > 25, with 1.8% of males and 3.8% of females in the category of BMI > 30.[2] In recent reports related to obesity and surgical operation, obesity was generally defined as a BMI > 30 in Western populations. However, we defined the obese group as having a BMI ≥ 28, because of the small number of Japanese people in the category of BMI > 30. The impact of laparoscopic colectomy for obese patients has been previously investigated. However, the indications and methods for laparoscopic colectomy varied greatly. Most studies on laparoscopic colectomy mentioned that this procedure was not intended for obese patients either with CRC or benign colon diseases, such as diverticulitis, although it was reported that the percentage of patients with CRC varied from 17.5% to 34 % among obese subjects.[5,9,10] In this study, the patients with CRC subjected to laparoscopic anterior resection were merely collected and retrospectively reviewed. Moreover, CRC location (S or RS) and degree of lymph node dissection (D2 or D3) were restricted to standardize the extent and quality of the operation.

In accordance with our operative data, there were no significant differences among the three groups in any parameter, including the number of ports (*P* = 0.284). Leroy *et al.*[9] reported that all obese patients (BMI > 30) were systematically subjected to laparoscopic left colectomy, using 6 ports to help maintain fatty mesentery and enlarged small bowel and mobilize the splenic flexure. As a result, the number of ports was the only significant intraoperative parameter associated with obesity in their study. Although we deemed that a larger number of ports could be used in obese patients to compensate for technical feasibility, we found no difference in the number of ports among the three groups. In our study, the length of the wound incision was the only significant intraoperative parameter associated with obesity. The rate of intraoperative complications was also similar in the obese, pre-obese and non-obese groups (11.1%, 27.3% and 35.6%, *P*=0.600). Although the criteria for intraoperative complications were not generally defined, our criteria included minor complications. Therefore, the percentages of intraoperative complications in this study were higher than those found in previous reports.[13,14] Intraoperative complications occurred in one patient in the obese group and three patients in the pre-obese group, but none of them required conversion to HALS or laparotomy.

In this study, the distribution of tumor invasion and stage were pathologically similar in the three groups. Moreover, the number of harvested nodes in the obese and preobese group was similar to that in the non-obese group. Leroy *et al*.[9] reported that the number of harvested nodes for CRC was similar in the obese and non-obese groups (7.2±6.4 and 9.1±5.5, respectively).There were no differences in the short-term outcome including the rate of conversion to HALS or laparotomy and that of postoperative complications and duration of postoperative hospital stay, among the three groups. The rate of re-operation was significantly different among the three groups, because there were two cases of incarcerated hernias in the port site in the pre-obese group. Tuech *et al.*[8] reported the outcome of laparoscopic colectomy for sigmoid diverticulitis. In their study, the patients were categorized in three groups; normal weight (BMI: 18.5-24.9), overweight (BMI: 25.0-29.9) and obese (BMI ≥ 30.0) groups. The operative time did not differ for the normal weight and the overweight groups (*P*=0.6), but it was shorter in the normal weight group than in the obese group (*P*=0.003). There were no differences in the conversion rate, morbidity rate or duration of hospital stay among the three groups. Although the indications for laparoscopic colectomy in obese patients differed from ours, their data were similar to our results.

With regard to postoperative complications, Senagore *et al.*[10] reported that anastomotic leakage was significantly increased in obese patients (5% vs. 1%, *P*<0.05). However, they noted that there were no reasons for a high anastomotic leakage rate in obese patients. Other investigators[5] found that the rate of wound infections was significantly higher in obese patients. They concluded that wound complications were unsolved issues in the postoperative management of obese patients, although subcutaneous drains[15] and retention sutures[16] were suggested to improve the outcomes of wound complications.

The duration of postoperative hospital stay was similar among the three groups. However, it was longer than that reported in Western countries, because most Japanese insurance plans cover the cost of hospitalization. As a result, the patients were not receptive to an early discharge, even if told and assured the postoperative course was good. Nevertheless, this has been gradually shortened by educating the patient and by improved clinical course.

In this study, the rate of postoperative complications was similar among the three groups. Yet, incarceration of the small bowel in a 10-mm port occurred in two patients in the pre-obese group and a new operation was necessary. Both of them were initial cases and after those cases we started closing all layers at 10-mm or larger port sites, including the peritoneum. Tonouchi *et al.* reviewed the literature regarding port site hernia and concluded that patients with port site hernias tended to have a higher BMI. Obese patients are at a high risk of developing preperitoneal hernias because of their substantially thicker preperitoneal space and elevated intra-abdominal pressure. Thus, port sites with a diameter of ≥ 10 mm should be closed completely, especially in obese patients.

## CONCLUSIONS

This retrospective study on laparoscopic colectomy for CRC demonstrated that operative time, blood loss, intraoperative complication rates and conversion rates were similar among obese, pre-obese and non-obese groups. In addition, postoperative morbidity rates and length of hospital stay did not differ among the groups. Laparoscopic colectomy can be performed safely in obese patients. It provides similar beneficial short-term outcomes not only in non-obese patients but also in pre-obese and obese patients.
